# The Quorum Sensing Volatile Molecule 2-Amino Acetophenon Modulates Host Immune Responses in a Manner that Promotes Life with Unwanted Guests

**DOI:** 10.1371/journal.ppat.1003024

**Published:** 2012-11-15

**Authors:** Arunava Bandyopadhaya, Meenu Kesarwani, Yok-Ai Que, Jianxin He, Katie Padfield, Ronald Tompkins, Laurence G. Rahme

**Affiliations:** 1 Department of Surgery, Harvard Medical School and Massachusetts General Hospital, Boston, Massachusetts, United States of America; 2 Department of Microbiology and Immunobiology, Harvard Medical School, Boston, Massachusetts, United States of America; 3 Shriners Hospitals for Children Boston, Boston, Massachusetts, United States of America; Yale University, United States of America

## Abstract

Increasing evidence indicates that bacterial quorum sensing (QS) signals are important mediators of immunomodulation. However, whether microbes utilize these immunomodulatory signals to maintain infection remain unclear. Here, we show that the *Pseudomonas aeruginosa* QS-regulated molecule 2-amino acetophenone (2-AA) modulates host immune responses in a manner that increases host ability to cope with this pathogen. Mice treated with 2-AA prior to infection had a 90% survival compared to 10% survival rate observed in the non-pretreated infected mice. Whilst 2-AA stimulation activates key innate immune response pathways involving mitogen-activated protein kinases (MAPKs), nuclear factor (NF)-κB, and pro-inflammatory cytokines, it attenuates immune response activation upon pretreatment, most likely by upregulating anti-inflammatory cytokines. 2-AA host pretreatment is characterized by a transcriptionally regulated block of c-JUN N-terminal kinase (JNK) and NF-κB activation, with relatively preserved activation of extracellular regulated kinase (ERK) 1/2. These kinase changes lead to CCAAT/enhancer-binding protein-β (c/EBPβ) activation and formation of the c/EBPβ-p65 complex that prevents NF-κB activation. 2-AA's aptitude for dampening the inflammatory processes while increasing host survival and pathogen persistence concurs with its ability to signal bacteria to switch to a chronic infection mode. Our results reveal a QS immunomodulatory signal that promotes original aspects of interkingdom communication. We propose that this communication facilitates pathogen persistence, while enabling host tolerance to infection.

## Author Summary

*Pseudomonas aeruginosa*, a recalcitrant Gram-negative opportunistic pathogen, defies eradication by antibiotics and exemplifies current highly problematic pathogens that often cause untreatable acute or chronic infections. There is increasing evidence that small molecules excreted by bacterial pathogens may impact human health by affecting functions including immunity. Although such small molecules in favor of acute infections have been reported, small molecules that may impact immune responses in pathogens' favor of maintaining a chronic infection have not. We recently published work showing that the *P. aeruginosa* small molecule 2-amino acetophenone (2-AA) promotes bacterial phenotypic and genetic changes associated with chronic infection. Here, we provide evidence that this diagnostically important small volatile molecule dampens the host inflammatory process triggered by infection, thus favoring chronic infection. 2-AA limits the inflammatory response by balancing the secretion of pro- and anti-inflammatory mediators *in vivo*, as well as *in vitro*, while improving survival against *P. aeruginosa* infection. The ability of 2-AA to dampen inflammatory processes, while increasing host survival and pathogen persistence, suggests that this molecule promotes host tolerance to infection. Our findings provide novel insights into pathogen weapons and mechanisms used to enable long-term bacterial presence in infected tissues.

## Introduction

Host-pathogen interactions are characterized by an antagonistic interplay between bacterial and host factors. The overall success of a pathogen depends on the efficacy of its virulence factors, anti-immune weapons, and the immune status of the host. Secreted microbial products, which include many virulence factors, play a critical role in the outcome of this antagonistic interaction. Bacterial quorum sensing (QS) regulates many of these products [1], [2]. QS is a communication system widely utilized by bacteria to perceive and promote collective behaviors that depend on population density signaling. This cell density-dependent communication system is achieved through the production and regulation of low-molecular-weight, excreted signal molecules [1], [3] as a means for pathogens to activate virulence factors [4] critical for infecting mammals [4]–[6]. Several such signal molecules are becoming more appreciated recently as important anti-immune weapons and mediators of inter-kingdom antagonistic relations [7].

One of the best characterized QS systems is that of the recalcitrant Gram-negative bacterium *Pseudomonas aeruginosa* [8], [9]. This opportunistic pathogen defies eradication by antibiotics and is an exemplar of bacteria that produce clinically problematic acute and chronic infections. It causes particularly difficult to treat infections in people with cystic fibrosis (CF), burn wounds, trauma, and compromised immune systems [10], [11]. *P. aeruginosa* controls the gene expression of many virulence factors through three QS systems: LasR, RhlR, and MvfR (PqsR) [9]. LasR and RhlR are activated by the N-acyl-homoserine lactone (AHL) signaling molecules N-3-oxododecanoyl homoserine lactone (3-oxo-C_12_-HSL) and N-3-butanoyl-Dl-homoserine lactone (C_4_-HSL) [9]. Meanwhile, MvfR (PqsR) is activated by the 4-hydroxy-2-alkylquinolines (HAQs) signaling molecules PQS (2-heptyl-3,4-dihydroxyquinoline (Pseudomonas Quinolone Signal) and HHQ (4-hydroxy-2-heptylquinoline) [5], [6], [8], [12]. In addition to their role as QS signal molecules, AHLs and HAQs also modulate inflammation and immune responses in mammals [7], [13]. The 3-oxo-C_12_-HSL signal molecule inhibits dendritic cell and T-cell activation [14], promotes apoptosis [15]–[17] and inhibits the ability of macrophages and monocytes to respond to a range of Toll-like receptor (TLR) agonists through disruption of nuclear factor (NF)-κB signaling [13]. 3-oxo-C_12_-HSL is a strong inducer of pro-inflammatory cytokines such as interleukin (IL)-6 and IL-8 in airway epithelial cells, lung fibroblasts, and macrophages, and is an enhancer of neutrophil chemotaxis [18]. Although it is known to upregulate inflammatory mediators through NF-κB [19] and extracellular regulated kinase (ERK)1/2 pathways [20], 3-oxo-C_12_-HSL does not activate the p38 mitogen-activated protein kinase (MAPK) pathway in lung epithelial cells [19]. Vikström *et al.* provided evidence that 3-oxo-C12-HSL specifically activates the p38 MAPK pathway in human macrophages, without activating the ERK1/2 signaling cascade [21]. Taken together, these data suggest that 3-oxo-C_12_-HSL suppresses key immune networks responsible for bacterial clearance, while simultaneously enhancing inflammatory pathways that promote *P. aeruginosa* pathogenesis. Relative to AHLs, HAQs have been investigated much less extensively. It has been reported that the HAQs signals PQS and HHQ do not affect apoptosis, but rather down-regulate host innate immune responses through the NF-κB pathway [22], [23]. Collectively, *P. aeruginosa* QS secreted small molecules serve multiple purposes in their effort to enhance bacterial pathogenesis and secure infection.

Immune response activation is a critical step in host resistance to infection and pathogen elimination. Perhaps the best studied defense signaling pathways are those that involve TLRs, which bind to microbial products, leading to the activation of innate immune responses critical for subsequent adaptive immune responses [24], [25]. Through a series of intracellular signaling molecules, microbial associated molecules activate NF-κB [26] and MAPKs, including ERK1/2, p38 kinase, and c-JUN N-terminal kinase (JNK) [27]. As regulators of several transcription factors, including NF-κB [28], [29], activator protein-1 (AP-1), and CCAAT/enhancer-binding protein (c/EBP), these kinases play an important role in initiating the expression of genes involved in immune responses [30], [31]. The rapid activation of diverse signaling pathways induces immune cells to express downstream genes encoding pro-inflammatory cytokines [32], which then alert the innate immune system. Such immune activation is required for pathogen elimination [33]–[36] However, microbial pathogens can actively inhibit activation of innate immune responses [37]–[39] thus favoring the establishment of a persistent lifestyle that may lead to chronic infection. Chronic infections are generally established following an acute infection period involving activation of both the innate and acquired immune systems [40]. During host tolerance—defined as coping with a pathogenic encounter without a consequent reduction in fitness [41]–[46] —the host's strategy is to avoid a harmful excessive inflammatory response [47], [48]. However, unfortunately for the host, this strategy may enable pathogen persistence.

Although recognition of the exploitation of host signaling pathways by QS regulated excreted molecules is increasing, it remains unclear whether pathogens employ QS to cause chronic infection and whether the host fights infection through the detection of these molecules. We showed recently that, through the control of the QS-regulated small volatile aromatic molecule 2-amino-acetophenon (2-AA) [49], the QS transcription factor MvfR promotes the emergence of *P. aeruginosa* phenotypes, thereby favoring pathogen adaptation and a chronic presence of *P. aeruginosa* [49]. 2-AA, which is responsible for *P. aeuriginosa's* sweet “grape-like” odor in culture and human infections [50], is one of the most abundant MvfR-controlled low molecular weight QS molecules produced and excreted by pathogen. This molecule has been proposed as a biomarker for *P. aeruginosa* colonization in burn wounds [50] and chronically infected CF lung tissues [51].

The presence of 2-AA in infected human tissues together with its ability to signal bacterial changes that favor chronic infection [49] raise the question of whether this molecule modulates host immune responses, and whether such modulation may favor the long-term presence of the pathogen. Thus, in the present study, we investigated 2-AA's possible immunomodulatory role. Animal and *ex vivo* studies were conducted to explore the impact of 2-AA on inflammatory processes as well as to assess 2-AA's effects on the activation of immune effectors and on the ability of the mice to tolerate the long-term presence of *P. aeruginosa*.

## Results

### 2-AA pretreatment leads to increased survival of mice and bacterial persistence in infected tissues

**Figure 1:**
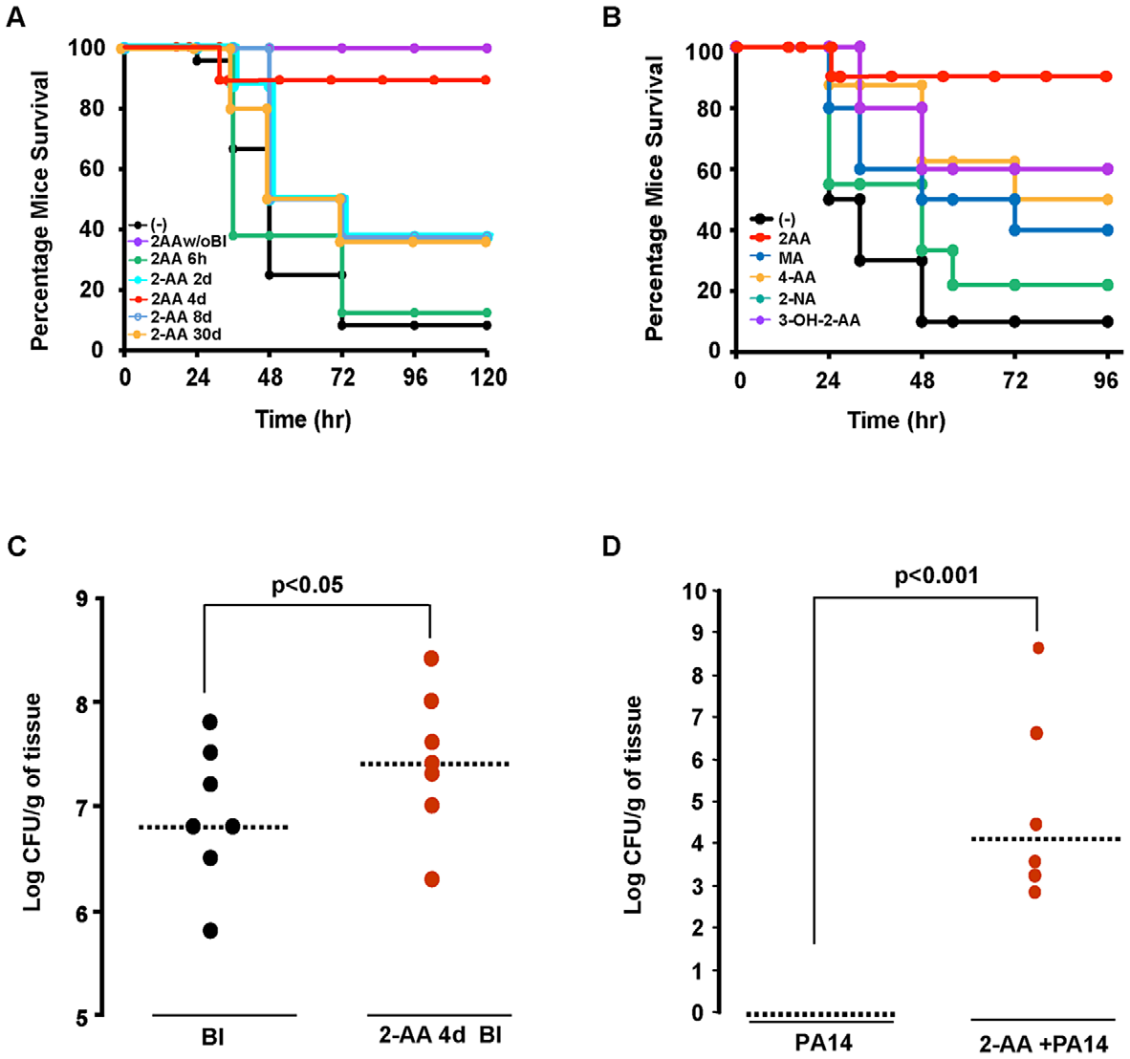
2-AA enhances survival following BI. (A) Mice were injected with 2-AA (6.75 mg/kg mice) or PBS 6 h (n = 20), 2 d (n = 20), 4 d (n = 20), 8 d (n = 20), or 30 d (n = 20) prior to BI with PA14. The data shown are averages of two independent experiments. Significance of survival rate differences was determined using the Kaplan-Meier method, with a hazard ratio of 1.8932 (95% CI, 1.0664–6.0718). Infection (−) drastically reduced survival relative to (2-AA W/O BI) controls (p = 0.03). Delivery of 2-AA 4 d before BI (red) had a particularly powerful influence on survival versus mice not pretreated with 2-AA (p = 0.03). A less remarkable, but still significant, survival benefit was also observed in BI mice pre-exposed to 2-AA 6 h, 2 d, 8 d, or 30 d before BI (all p = 0.03 vs. non-infected 2-AA exposed controls). (B) Relative to the effects observed with 2-AA (n = 20), 4 d pretreatment with the 2-AA analogs 4-AA (n = 8; p = 0.03), 2-NA (n = 8; p = 0.03), or MA (n = 8; p = 0.03), or the 2-AA metabolite 3OH-2-AA (n = 8; p = 0.03) prior to PA14 infection had weak, though still statistically significant, positive effects on survival after infection. Significance of survival rate differences was calculated as in A. (C) Bacterial loads in the local muscle 7 d post-BI were significantly higher in mice pretreated with 2-AA 4 d before BI (n = 7) than in control mice subjected to BI without 2-AA pretreatment (n = 7; p<0.05, Kruskal-Wallis test). CFU data are presented on a log_10_ scale. (D) CFU counts at the site of infection in mice 11 d postinfection. The 2-AA treated mice showed proliferation and higher counts than mice that were not treated with 2-AA. (n = 6; p<0.001, Kruskal-Wallis test). CFU data are presented on a log_10_ scale.

To determine if 2-AA modulates host immune responses, we assessed the susceptibility of 2-AA pretreated mice to *P. aeruginosa* infection (strain PA14) using a burn and infection (BI) model [52]. We observed a time-interval dependent protective effect of host pre-exposure to 2-AA. Mice pretreated with 2-AA 6 h or 1 d pre-BI showed no protection; the animals died at the same rate as untreated BI controls (Fig. 1A). However, mice injected 4 d pre-BI had a survival rate of 90%, which was remarkably better than the 10% rate observed in untreated BI controls (Fig. 1A). Pretreatment with 2-AA 2 d, 8 d, or 30 d before BI had more modest benefits; these mice showed survival rates of 50%.

Animals pretreated with the 2-AA metabolite 3OH-2-AA [53] 4 d prior to BI (Fig. 1B) showed a lesser degree of protection (60% survival at 72 h) relative to animals pretreated with 2-AA, indicating that the robust effect observed with 2-AA could not be attributed to the 3OH-2-AA metabolite. Pretreatment with the 2-AA structural analogs 4-amino acetophenone (4-AA), 2-nitro-acetophenone (2-NA), or methyl anthranilate (MA) (Fig. S2) provided a moderate level of protection (20–50% survival when delivered 4 d before BI) relative to that observed with 2-AA (90%) (Fig. 1B). These findings show that 2-AA provides markedly stronger protection than structurally similar molecules, indicating that the 2-AA effect is relatively specific.

The survival benefit yielded by 2-AA pretreatment cannot be attributed to the reduced bacterial proliferation since 2-AA pretreatment significantly increased PA14 loads at the wound site 7 d after BI compared to untreated controls (Fig. 1C). Moreover using a chronic wound infection model [54], we showed that animals co-inoculated with 2-AA and bacteria maintained high PA14 loads (mean of ∼1×10^4^ colony forming units [CFU]/g of tissue) in infected tissues 11 d post-infection, whereas the untreated animals almost completely cleared the bacteria (Fig. 1D). These results suggest that 2-AA supports the long term presence of bacteria.

### 2-AA modulates the inflammatory response *in vivo*

**Figure 2:**
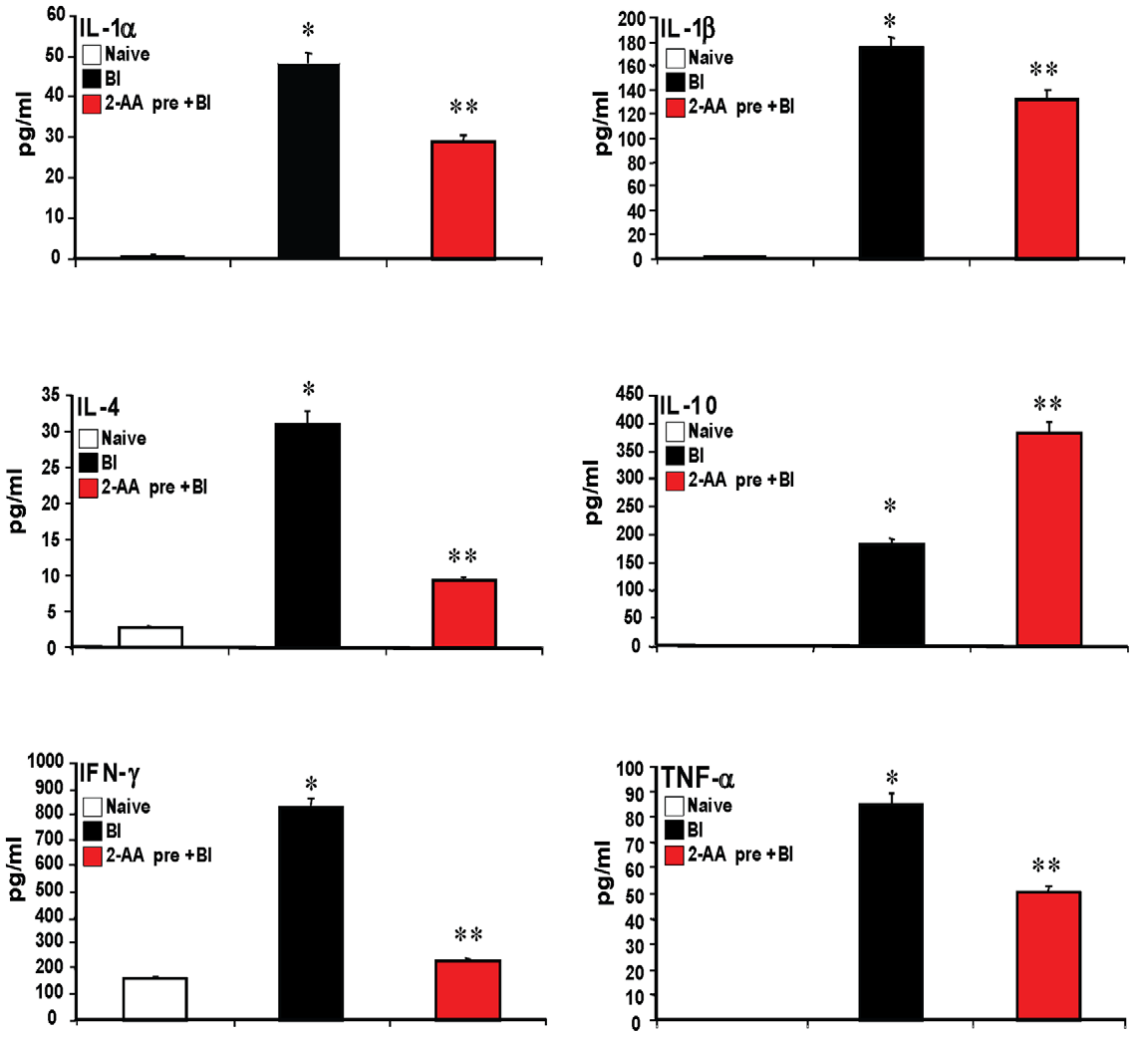
2-AA pretreatment modulates the pro-inflammatory response *in vivo*. Multiplex ELISA showed that BI induced surges in serum levels of IL-1α, IL-1β, IL-4, IL-10, IFN-γ, and TNF-α 24 h post-BI, and that 2-AA pretreatment delivered 4 d before BI attenuated the surges in IL-1α, IL-1β, IL-4, IFN-γ, and TNF-α, while augmenting the surge in IL-10 (n = 4 per group). Mean values calculated from 2–4 replicate experiments are depicted with SD error bars. *p<0.05 vs. naïve; **p<0.05 vs. BI (Student's t test).

We proceeded to examine the mechanism by which 2-AA can reduce mortality against *P. aeruginosa* without eliminating bacterial load in mice. We found that 2-AA treatment 4 d pre-BI reduced serum levels of the pro-inflammatory cytokines IL-1α, IL-1β, IL-4, interferon (IFN)γ, and tumor necrosis factor (TNF)-α, compared to untreated BI mice, while augmenting secretion of the anti-inflammatory cytokine IL-10 (Fig. 2). No effects of the pretreatment on IL-6, IL-2, and IL-12 were observed (data not shown). These results suggest that 2-AA pretreatment may reduce inflammation by maintaining a balance between pro- and anti-inflammatory processes.

**Figure 3:**
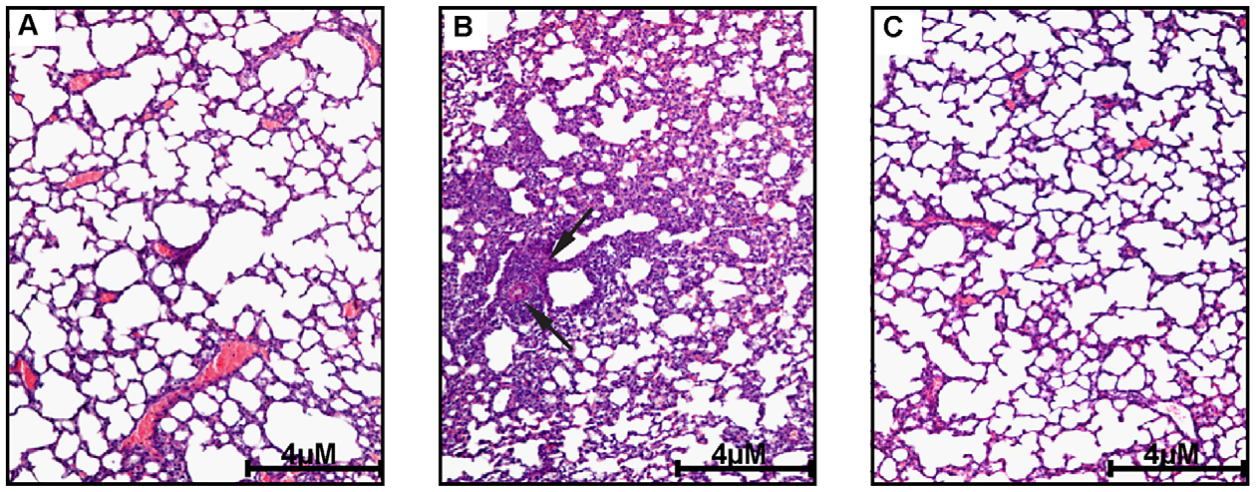
Histopathology of lung tissues after 2-AA treatment. (A) Control healthy (non-infected) lung tissue 4 d after 2-AA treatment. (B) Inflammatory cell infiltration with large areas of consolidation in lung parenchyma 48 h after infection with PA14 (Black arrows indicate the infiltration and necrotic foci). (C) Lack of infiltration 48 h after PA14 infection in the lungs of mice pretreated with 2-AA 4 d prior to BI.

Additionally, we found that pretreating mice with 2-AA 4 d prior to infection provided protection against severe inflammation in an acute lung infection model [55]. Lung histopathology was compared with untreated control mice (Fig. 3A). Untreated mice infected with PA14 rapidly developed extensive inflammation within the lungs, as evidenced by a red hepatization of the lung tissues 24 h after infection. After 48 h, there was extensive progression of the pneumonia with the formation of bacteria-filled necrotic foci throughout the lung parenchyma (Fig. 3B). In sharp contrast, lung inflammation was markedly attenuated in mice given 2-AA 4 d prior to being infected (Fig. 3C).

### 2-AA pretreatment represses 2AA-induced NF-κB activation pathway in mouse macrophages

**Figure 4:**
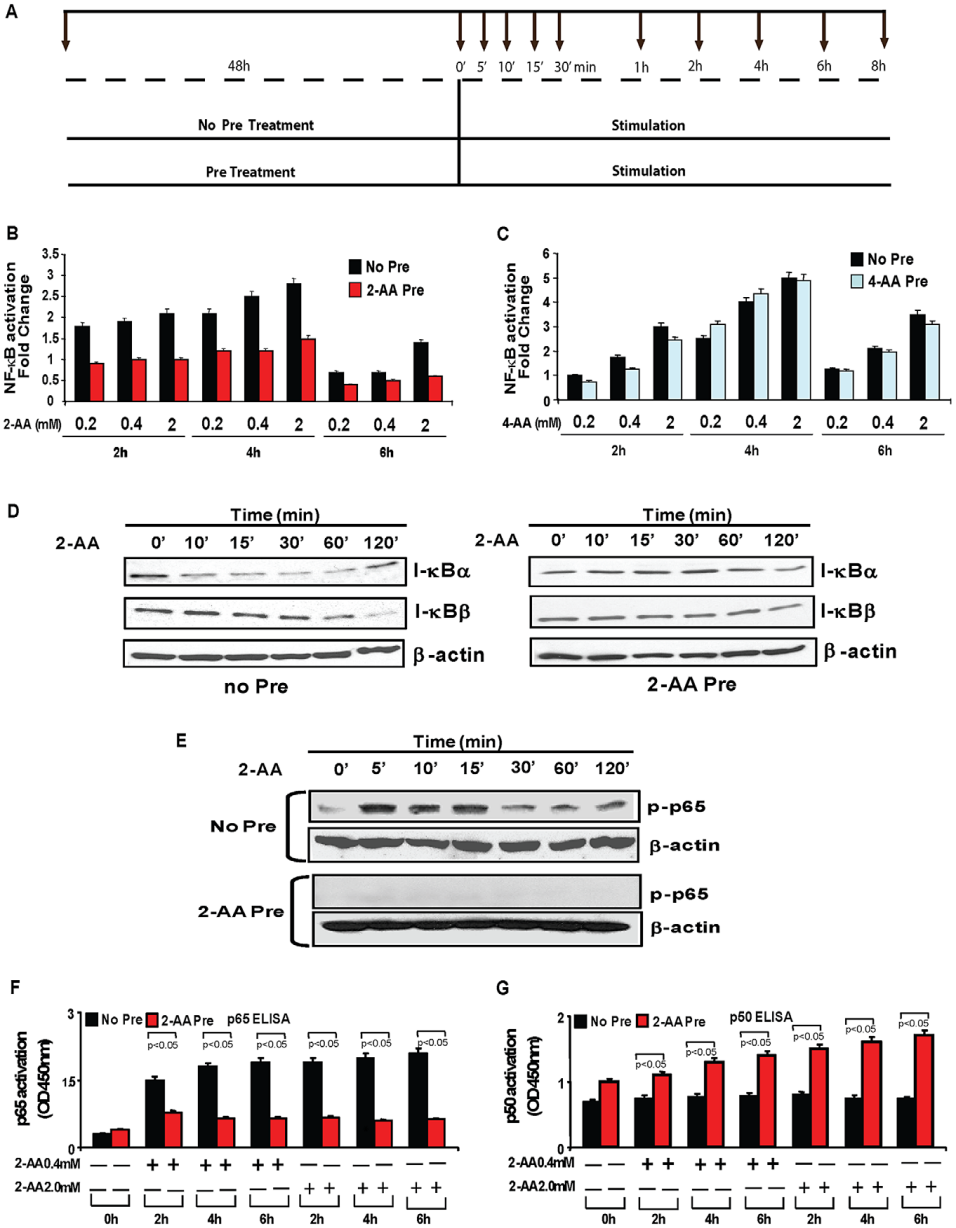
2-AA pretreatment modulates activation of the NF-κB pathway in mouse macrophages. (A) Schematic of 2-AA treatment. Macrophages were left untreated (No Pre) or pretreated with 0.8-mM 2-AA or 4-AA for 48 h (2-AA/4-AA Pre). The untreated and 2-AA pretreated cells were then stimulated with 0.2 mM, 0.4 mM, or 2.0 mM 2-AA (for experiment in B) or 4-AA (for experiment in C). (B) Pretreatment with 2-AA blocked NF-κB activation relative to cells not pretreated with 2-AA (0.8 mM). (C) NF-κB was activated by 2-AA analog 4-AA in 4-AA pretreated and not pretreated cells. Mean values calculated from 2–4 replicate experiments are depicted with SD error bars. (D and E) Following stimulation with 2-AA (0.4 mM), cellular extracts prepared from not pretreated and 2-AA pretreated macrophages. Western blots of I-κBα and I-κBβ degradation (D) and phosphorylation of NF-κB subunit p65 (E). Loading was normalized relative to mouse β-actin. (F and G) A TransAM NF-κB assay showed binding of NF-κB p65 and p50 with the NF-κB promoter in not pretreated and 2-AA pretreated cells following stimulation with 2-AA. Mean values calculated from three replicate experiments are depicted with SD error bars. (p<0.05, Student's t test).

We examined how 2-AA affects the innate immune system *ex vivo* in a mouse macrophage cell line stably expressing a NF-κB-luciferase transcriptional fusion gene. 2-AA stimulation produced a dose-dependent, transient NF-κB activation that peaked at about 4 h (Figs. 4A–B, S1A). Interestingly, this NF-κB activation effect of 2-AA stimulation was dampened in macrophages pretreated with 2-AA for 48 h (Fig. 4B). This dampening effect was not due to 2-AA cytotoxicity (see data in Fig. S3). Furthermore, the effect was found to be related to 2-AA since pretreatment of macrophages with the 2-AA analogs 3-amino acetophenone (3-AA) (data not shown) and 4-AA (Fig. 4C) did not modify 2-AA induced NF-κB activation.

Stimulation of macrophages with 2-AA caused a rapid degradation of the NF-κB inhibitor I-κBα within 15 min, and cleavage of the NF-κB inhibitor I-κBβ by 60–120 min. In contrast, 2-AA-pretreated macrophages maintained high levels of I-κBα and I-κBβ upon stimulation (Fig. 4D). Western blot analysis further showed that, in 2-AA pretreated cells, phosphorylation of the p65 subunit of NF-κB, which enables I-κBα release and proteolysis [56], was reduced relative to that in non-pretreated cells (Fig. 4E & S5A) Additionally, a concentration-dependent increase in DNA binding of activated NF-κB p65 was observed in non-pretreated macrophages upon 2-AA stimulation, whereas this DNA binding was reduced in 2-AA pretreated cells (Fig. 4F). In contrast, p50 binding was increased in 2-AA pretreated cells (Fig. 4G). Taken together, these findings demonstrate that 2-AA pretreatment represses NF-κB activity. As shown in Figure S4, NF-κB activity was also dampened in 2-AA pretreated macrophages stimulated with other pathogen-associated molecules, such as LPS and peptidoglycan (PGN), providing additional support for the validity of our observations with 2-AA.

### 2-AA pretreatment down-regulates pro-inflammatory cytokines while upregulating anti-inflammatory cytokine production in mouse macrophages

**Figure 5:**
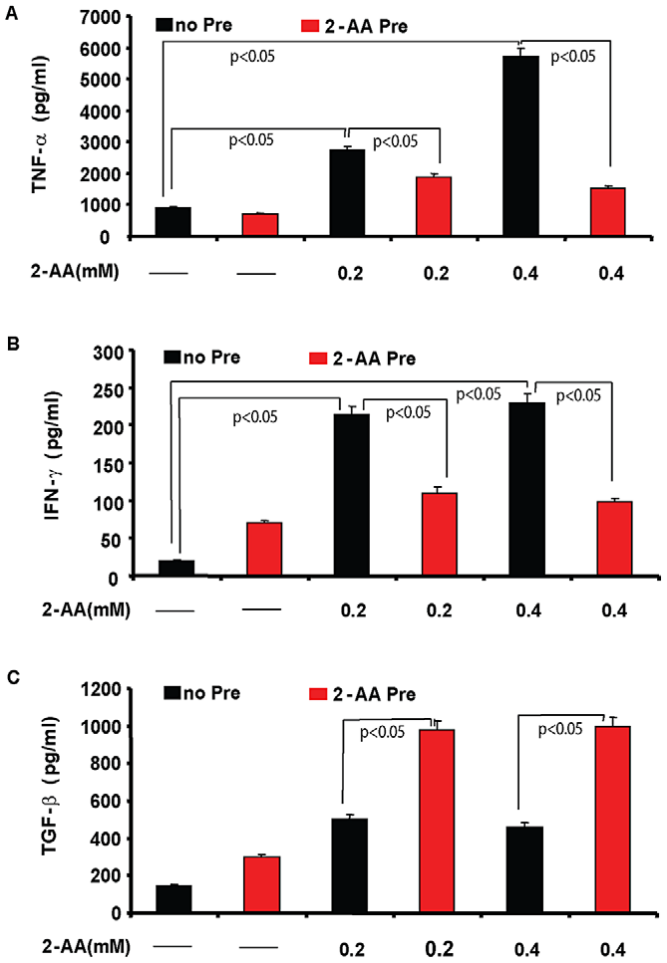
2-AA pretreatment alters the expression of pro- and anti-inflammatory cytokines upon 2-AA stimulation in macrophages. Levels of TNF-α (A), IFN-γ (B), and TGF-β (C), following 6 h stimulation of 2-AA as measured by ELISA. The experiments were performed in triplicate and the results are expressed as means ± SD. (p<0.05, one-way ANOVA).

Since NF-κB regulates the production of pro-inflammatory mediators [57], we hypothesized that 2-AA pretreatment would reduce the production of pro-inflammatory cytokines, as seen in the *in vivo* experiments presented above (Fig. 2). Compared to 2-AA non-pretreated macrophages, 2-AA pretreated cells showed a decreased production of the pro-inflammatory cytokines TNF-α and IFN-γ (Fig. 5A and 5B), and an increased release of the anti-inflammatory cytokine TGF-β following stimulation with 0.2 or 0.4 mM 2-AA (Fig. 5C).

### 2-AA pretreatment modulates MAPK signaling components of innate immunity

**Figure 6:**
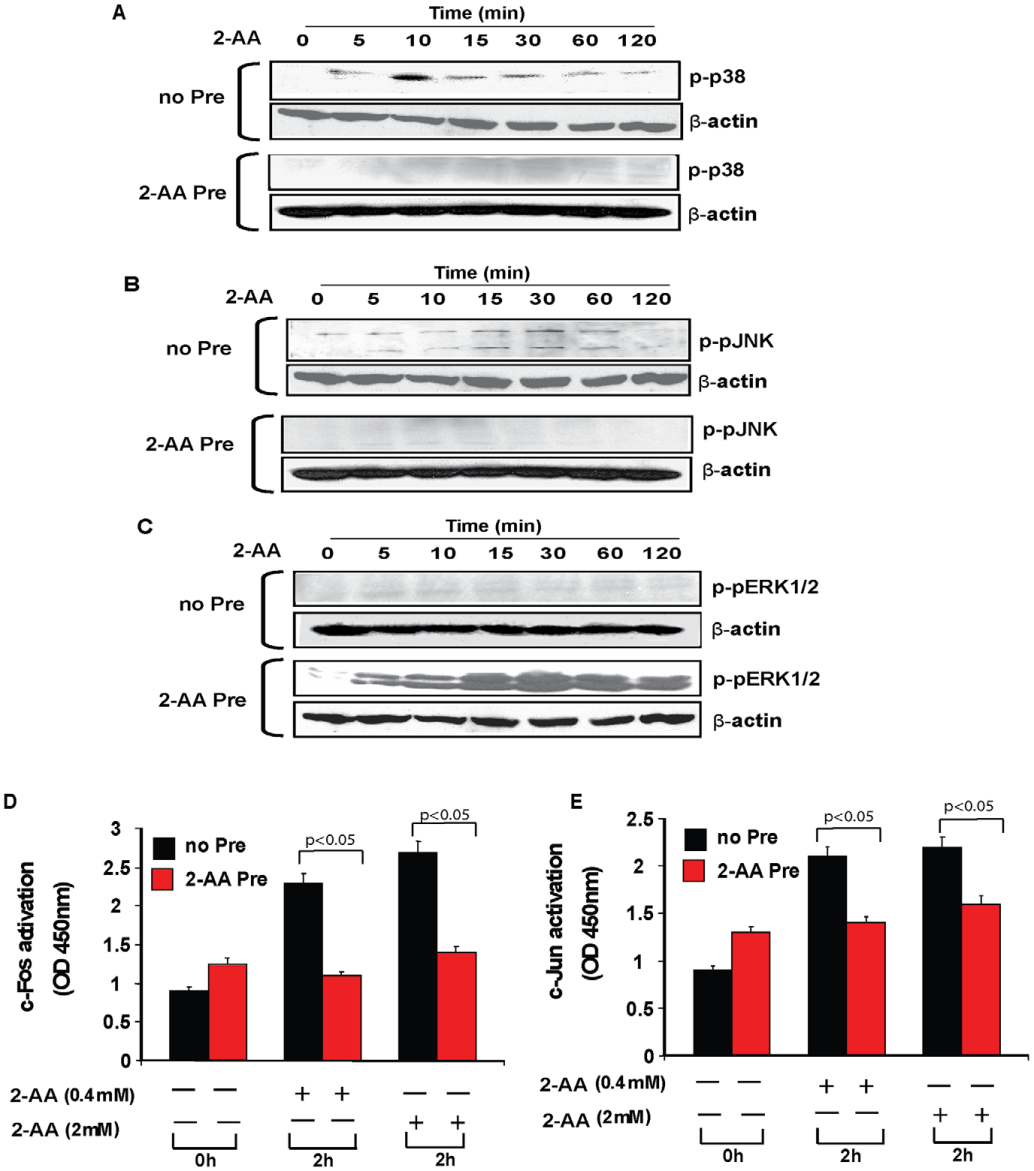
2-AA pretreatment alters activation of the MAPKs and AP-1 in macrophages upon 2-AA stimulation. Western blotting of cellular extracts with phospho-specific antibodies after 48 h pretreatment with 2-AA (0.8 mM) followed by stimulation with 0.4 mM 2-AA (A) p38 MAPK, (B) JNK1/2 and (C) ERK1/2. One representative experiment (out of three) is shown. Loading was normalized relative to mouse β-actin. A TransAM AP-1 transcription factor assay after 48-h pretreatment with 2-AA (0.8 mM) followed by stimulation with 2-AA, showing the binding of c-Fos (D) and c-Jun (E) to AP-1 promoter. Mean values calculated from three replicate experiments are depicted with SD error bars. (p<0.05, Student's t test).

We explored the effects of 2-AA stimulation and pretreatment on activation of ERK1/2, JNK, p38 MAPK, c-Jun, and c-Fos, which are essential components of innate immune signaling pathways in macrophages [58], [59]. A transient increase in phosphorylated p38 and JNK1/2 MAPKs was observed 5–15 min after 2-AA stimulation (Fig. 6A & B, S5B & C). This increase was blocked in 2-AA pretreated macrophages (Fig. 6A & B, S5B & C). 2-AA stimulation did not activate ERK1/2 in 2-AA naïve cells, but did induce ERK1/2 phospholyration in 2-AA pretreated cells (Fig. 6C & S5D).

TransAM assays demonstrated that 2-AA stimulation of 2-AA naïve cells resulted in phosphorylation, and therefore activation, of the AP-1 family transcription factors c-Fos and c-Jun, enabling them to bind to the AP-1 promoter element. This 2-AA stimulation-induced binding of c-Fos and c-Jun was dampened in pretreated cells (Fig. 6D & E). These data fit with the aforementioned down-regulation of JNK and p38 phosphorylation observed in 2-AA pretreated macrophages. Pretreatment with the 2-AA analog 4-AA did not alter c-Fos and c-Jun binding to the AP-1 element (Fig. S6).

### c/EBPβ over-expression and formation of the c/EBPβ-p65 complex supports the inhibition of p65 phosphorylation in 2-AA pre-treated cells

**Figure 7:**
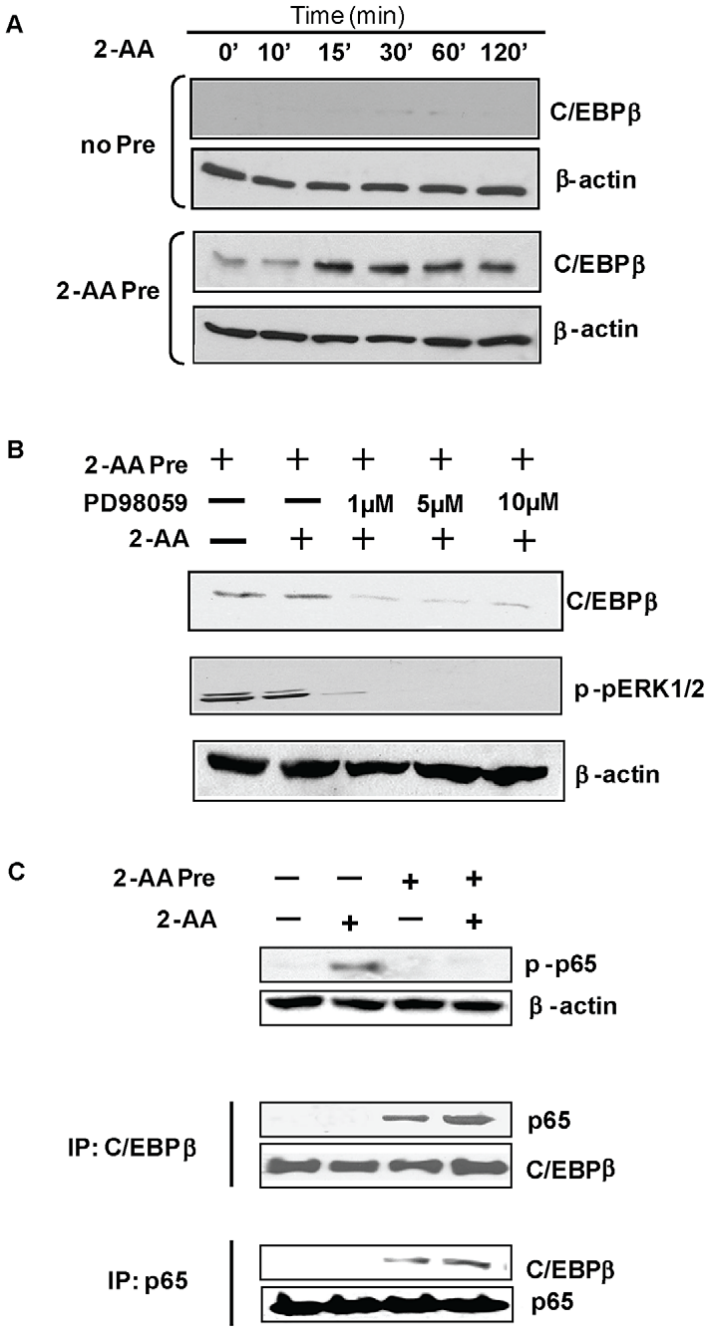
Inhibition of p65 phosphorylation in 2-AA pretreated cells is accompanied by *de novo* formation of c/EBPβ-p65 complexes. (A) Western blots of cellular extracts incubated with c/EBPβ from macrophages that had been incubated for 48 h with 0.8 mM 2-AA (2-AA Pre) or plain medium (No Pre) and subsequently stimulated with 0.4 mM 2-AA for the indicated time periods. (B) Western blot showing inhibition of ERK1/2 and c/EBPβ in 2-AA pretreated cells in the presence of MEK1 inhibitor PD98059 (1 µM, 5 µM, or 10 µM). Loading was normalized relative to mouse β-actin. (C) In cells treated as above, c/EBPβ-p65 complex formation monitored by IP followed by immunoblotting with anti-c/EBPβ or anti-p65 antibodies.

To investigate the possible role of ERK1/2 in dampening of inflammation after 2-AA pretreatment, we examined c/EBPβ and NF-κB activation [60]. We found that expression of the c/EBPβ, which functions downstream of the MEK-ERK1/2 pathway [60], was markedly increased in 2-AA pretreated macrophages following 2-AA stimulation relative to the expression observed in non-pretreated, stimulated macrophages (Fig. 7A). Moreover, in the presence of a MEK inhibitor, c/EBPβ expression was blocked and 2-AA pretreated cells showed the expected down-regulation of ERK1/2 and c/EBPβ (Fig. 7B).

We proceeded to examine whether increased c/EBPβ expression contributes to the prevention of NF-κB activation in 2-AA pretreated cells. As shown in Figure 7C, 2-AA stimulation resulted in phosphorylation of the NF-κB p65 subunit at ser 536 in the trans-activating domain (TAD)-1 in non-pretreated cells, but this phosphorylation was abolished in 2-AA pretreated cells. Moreover, co-immunoprecipitation (IP) studies supported the notion that c/EBPβ/p65 complex formation occurs in 2-AA pretreated cells only (Fig. 7C). Formation of the c/EBPβ-p65 complex prevents subsequent p65 activation.

### 2-AA-mediated silencing is controlled, at least in part, at the transcriptional level

**Figure 8:**
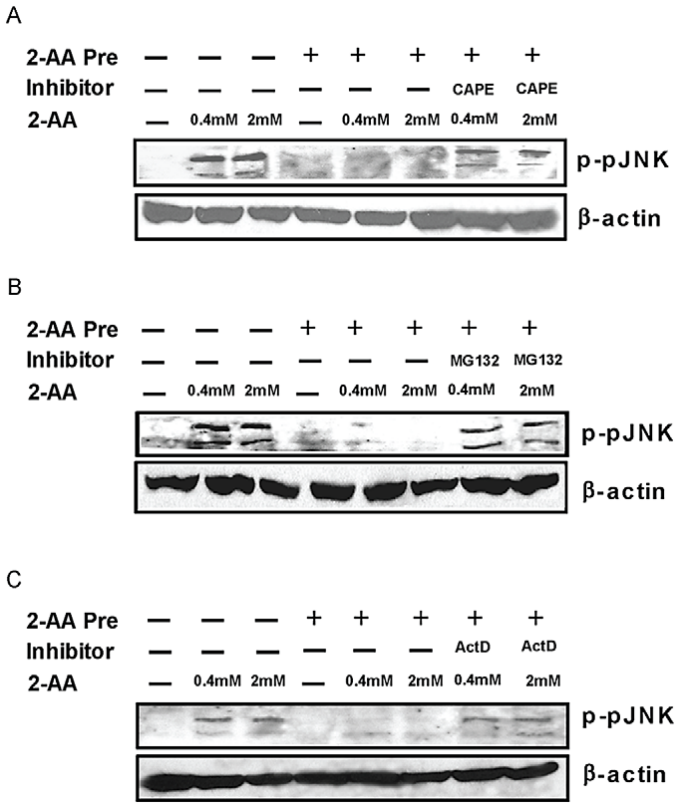
Transcription and NF-κB inhibitors can block the effects of 2-AA pretreatment. Western blots showing phosphorylation of JNK1/2 in 2-AA pretreated or untreated cells along with CAPE (1.5 µM) (A), MG-132 (1 µM) (B), and actinomycin D (1 µM) (C) following 0.2 mM or 0.4 mM 2-AA stimulation. Loading was normalized relative to mouse β-actin. One representative experiment (of three) is shown for JNK1/2.

Because 2-AA stimulation alone caused activation of NF-κB and transcriptional targets of NF-κB can inhibit JNK activation [59], we used NF-κB signaling inhibitors, at the time of 2-AA pretreatment, to examine the possibility that activation of NF-κB may account for 2-AA mediated silencing. We found that JNK phosphorylation was sustained after stimulation with 2-AA in the presence of NF-κB inhibitors (Fig. 8A & B). Further exposure of macrophages to the transcription inhibitor actinomycin D at the time of 2-AA pretreatment also partially restored phosphorylation of JNK in response to subsequent 2-AA stimulation (Fig. 8C), suggesting that the pretreatment-induced molecular silencing effect observed is controlled, at least in part, at the transcriptional level.

## Discussion

This study demonstrates the contribution of the low-molecular-weight QS molecule 2-AA in the modulation of mammalian innate-immune signaling pathways. This is the first study to show that this long-known [61], but scarcely studied, *P. aeruginosa* molecule modulates host responses. Unlike other immune-suppressing QS molecules, which promote activation of virulence functions leading to acute infection [8], [62], herein we showed that 2-AA minimizes activation of immune effectors and increases survival of infected animals, while permitting a long-term presence of the pathogen *in vivo*. Our cytokine profiling results suggest that 2-AA pretreatment limits inflammation by dampening pro-inflammatory cytokine activation. These data support the notion that 2-AA pretreatment protects host animals by balancing pro- and anti-inflammatory cytokine levels. 2-AA's ability to dampen host inflammation may be critical for both host survival and long-term survival of 2-AA secreting bacteria in host tissues.

Chronic infection is normally established after an acute infection period involving activation of both the innate and acquired immune systems. The ability of a host to tolerate a bacterial presence without negatively affecting the pathogen's fitness is particularly important since 2-AA eventually decreases the expression of many acute *P. aeruginosa* virulence related genes [49], making the pathogen vulnerable to clearance. Importantly, 2-AA not only silences the MvfR virulence regulon [49], but also promotes bacterial changes that favor long-term survival in a dynamic host environment. Thus, considered together with our prior published findings, the present results strongly suggest that 2-AA may serve a dual purpose: (1) to promote bacterial changes that favor chronic adaptability of the pathogen and (2) to suppress innate immune responses, allowing successful bacterial maintenance in host tissues.

The 2-AA mediated concomitant regulation of pro-inflammatory and anti-inflammatory cytokines resembles the endotoxin tolerance promoted by lipolysaccharide (LPS) [63], [64]. The decreased mortality observed in LPS-pretreated animals, however, is accompanied by a more efficient bacterial clearance [65], [66], rather than a long-lasting bacterial presence as occurred in our 2-AA-pretreated animals. Recently, Khajanchi *et al.* showed that animals pretreated with the QS molecule 3-oxo-C_12_-HSL had reduced levels of pro-inflammatory cytokines and cleared *Aermonas hydrophila* bacteria without tissue damage [67]. Conversely, we found that 2-AA pretreatment not only did not clear *P. aeruginosa* bacteria or affect its growth, but rather resulted in a higher bacterial load relative to that in non-pretreated mice. These findings strongly suggest that 2-AA's effect on host responses may enable the long-term presence of *P. aeruginosa*. A similar phenomenon was observed in plant infections involving the virulence factor XopD, which promotes *Xanthomonas campestris* pathovar *vesicatoria* growth *in planta*, while reducing host defense responses and delaying the development of disease symptoms [68].

**Figure 9:**
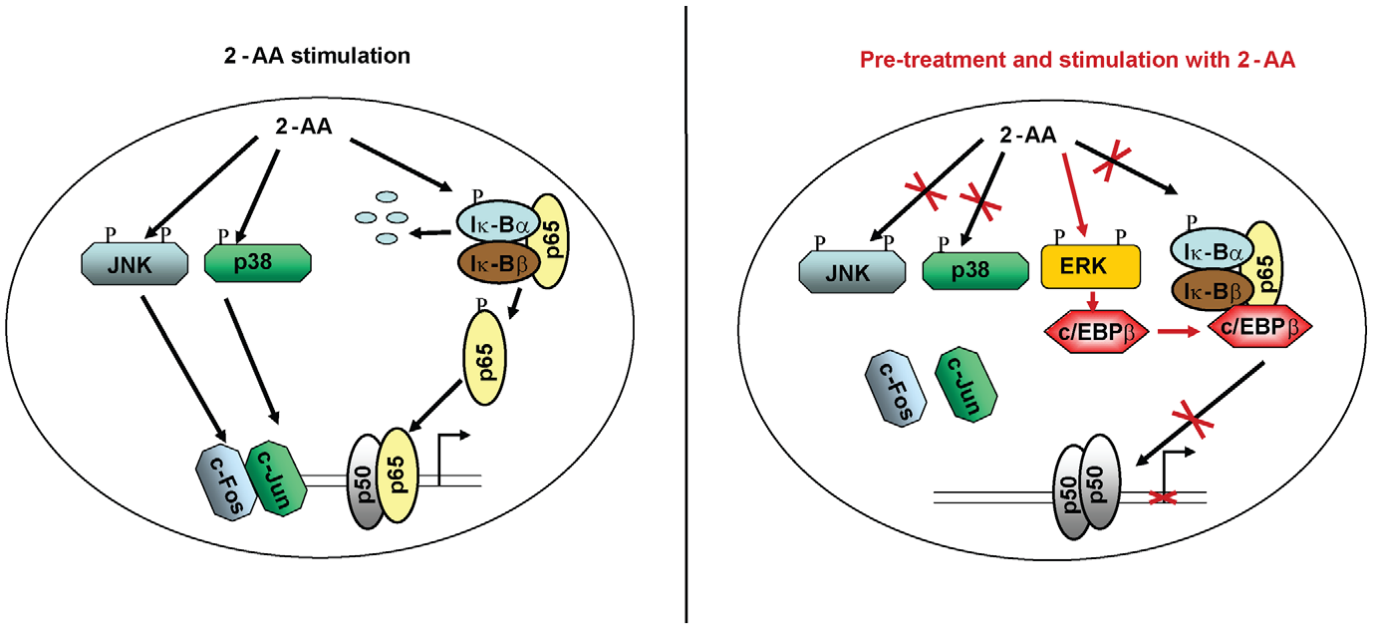
Proposed model for 2-AA immunomodulatory mechanisms. In naïve cells (left), stimulation with 2-AA induces activation of NF-κB, which leads to the phosphorylation and degradation of I-κBα, releasing the NF-κB dimers p65 and p50. 2-AA also induces the p38 MAPK and JNK pathways to stimulate c-Jun and c-Fos. Activation of MAPK and NF-κB pathway upregulates pro-inflammatory genes. In contrast, in 2-AA pretreated cells (right) over-expression of ERK1/2 activates C/EBPβ, which binds directly to p65, resulting in c/EBPβ-p65 complex formation, and preventing 2-AA induced phosphorylation of p65 upon 2-AA stimulation. This interaction inhibits NF-κB mediated transactivation. The activation of JNK and p38 MAPK are repressed in 2-AA pretreated cells. All together, repression of the p38 MAPK, JNK, and NF-κB pathways abrogates the activation of pro-inflammatory mediators.

Several recent studies have shown down-regulation of NF-κB, ERK1/2, JNK, and p38 MAPK activation in endotoxin-tolerant mouse macrophages [63], [69]. Although clarifying the exact molecular mechanism mediating the 2-AA pretreatment response will require further investigation, our findings show that 2-AA negatively impacts activation of NF-κB, JNK, and p38 MAPK, but increases ERK activation in pretreated cells. Based on the presented findings, we propose the model depicted in Figure 9. Briefly, 2-AA pretreatment may trigger a tolerance phenomenon to subsequent 2-AA challenges, leading to a dramatically increased survival rate of *P. aeruginosa* infected mice. Our findings further suggest that these effects are achieved by a block in MAPK and NF-κB activation, and activation of ERK1/2, leading to c/EBPβ activation and formation of the c/EBPβ-p65 complex that prevents NF-κB activation. In support of this model, we found that 2-AA strongly increased p65 phosphorylation in non-pretreated macrophages, but not in 2-AA pre-treated cells. It is well known that pro-inflammatory stimuli induce phosphorylation of NF-κB subunit p65, which is thought to increase the transactivation potential of p65 [70]–[72] and that suppression of p65 phosphorylation coincides with inhibition of I-κBα degradation [56]. Moreover, we showed that pretreatment with 2-AA induces the expression of ERK1/2 and in turn c/EBPβ, which binds directly to p65, resulting in c/EBPβ-p65 complex formation. ERK1/2 induction down-regulates NF-κB mediated transcription [29] and initiates post-transcriptional modification of c/EBPβ. This modification leads to a conformational change in c/EBPβ that unmasks its bZIP heterodimerzation domain [30], thereby enabling formation of the c/EBPβ-p65 complex [73], [74]. Formation of the c/EBPβ-p65 complex may mechanically alter interactions between NF-κB p65 and its inhibitors by blocking specific phosphorylation sites [75], [76], thereby enabling c/EBPβ to form an inhibitory box [77] in 2-AA pretreated cells. Since c/EBPβ is involved in immune modulation [31], c/EBPβ-p65 association in 2-AA pretreated cells could result in reduced expression of pro-inflammatory cytokines.

Interestingly, LPS pretreated cells also exhibit reduced NF-κB activation and binding activity, accompanied by decreased degradation of IκBα and IκBβ [63]. Thus, as shown in Figure 9, a 2-AA induced tolerance in murine macrophages *ex vivo* may be achieved by inhibiting activation of both NF-κB and AP-1 factors (i.e. c-Jun and c-Fos). Additionally, our observations that actinomycin D and NF-κB inhibitors can block 2-AA pretreatment effects on JNK activation are consistent with the idea that 2-AA works at the transcriptional level and requires *de novo* protein synthesis. We also showed that 2-AA pretreated macrophages were not fully responsive to other pathogen-associated molecules, such as LPS and peptidoglycan (PGN) (Fig. S4). Therefore dampening of immune signaling pathways by other pathogen associated molecules [78], [79] or endogenous danger signals—such as pro-inflammatory cytokines [80], heat shock proteins [81], [82], and hayluran [83]—might also play a role in infection-induced immunosuppression and the establishment of chronic/persistent bacterial infections. It remains to be determined whether these other agents also improve host survival *in vivo* and modulate defense mechanisms in the context of an active infection.

Several mechanisms may collectively contribute to 2-AA mediated tolerance *in vivo* and *ex vivo*. Firstly, genes encoding pro-inflammatory cytokines are inactivated to limit tissue damage. Meanwhile, anti-inflammatory mediators that do not disrupt host tissue physiology provide protection from inflammation and pathogen-induced damage. Our data indicate that selective and transient inactivation of immune mediators at the intracellular signaling level may play an important role in 2-AA induced host tolerance.

In conclusion, our results strongly support the notion that 2-AA produces immunomodulatory effects that enhance the host's ability to tolerate a pathogen presence. Meanwhile, 2-AA silences bacterial acute virulence functions [49] and dramatically increases survival rates among infected mice (Fig. 1A). From an evolutionary perspective, it is intriguing that *P. aeruginosa* would produce a QS molecule that decreases its own virulence. However, this seemingly counter-intuitive effect can ultimately be adaptive if it enables the pathogen to secure long-term survival within its host. This hypothesis is supported by data showing that 2-AA pretreatment ameliorated the inflammatory response upon subsequent infection, allowing infected mice to survive, and thus increasing the pathogen's fitness. 2-AA's ability to render immune cells tolerant, as observed in the present study, may mirror the mechanism by which this pathogen avoids elimination and persists in chronic infections. Elucidation of the molecular mechanisms involved in enabling host organisms to tolerate persistent/chronic infections may open new avenues for the development of diagnostics and therapeutic strategies to treat chronic, persistent, and relapsing infections.

## Materials and Methods

### Ethics statement

This study was carried out in strict accordance with the recommendations of the Guide for the Care and Use of Laboratory Animals of the National Institutes of Health. The protocol was approved by the Committee on the Ethics of Animal Experiments at Massachusetts General Hospital (Permit Number: 2006N000093/2, for burn wound infection and 2005N000111, for open wound infection). All procedures were performed under ketamine/xylazine anesthesia, and every effort was made to minimize suffering.

### Bacterial strains and growth conditions

A *P. aeruginosa* strain known as Rif^R^ human clinical isolate UCBPP-PA14 (commonly referred to as PA14) was used in the present experiments [84], [85]. The bacteria were grown at 37°C in Luria-Bertani (LB) broth under shaking and aeration or on plates of LB agar containing appropriate antibiotics, unless otherwise indicated. Overnight PA14 cultures were grown in LB+rifampicin (50 mg/L) and diluted the following day in fresh LB media.

### Mouse mortality and CFU assays

A thermal injury mouse model [52] was used as described previously [85] to assess bacterial pathogenicity in 6-wk-old CD1 mice (Charles River; Boston, MA). Following administration of anesthesia, a full-thickness thermal burn injury involving 5–8% of the total body surface area [52] was produced on the dermis of the shaved mouse abdomen, and an inoculum of 5.0×10^5^ PA14 cells in 100 µl of saline was injected intradermally into the burn eschar. Mouse survival was subsequently assessed over the course of 10 d.

2-AA treated mice received a single intravenous (IV) injection of 2-AA (6.75 mg/kg mice) 6 h, 2 d, 4 d, 8 d, and 30 d prior to BI. Mice treated with 4-AA (6.75 mg/kg mice), 2-NA (8.25 mg/kg mice), MA (7.55 mg/kg mice), 3OH-2-AA (7.6 mg/kg mice), and/or phosphate buffered saline (PBS; vehicle control) also received a single IV injection, but 4 d prior to BI. All injected compounds were purchased from Sigma-Aldrich (Saint Luis, MO).

To allow examination of bacterial persistence in the BI model, we inoculated mice with 2×10^3^ PA14 cells to avoid mortality of the control set. Animals were injected with 2-AA (6.75 mg/kg mice) 4 d prior to BI. After 4 d, 2×10^3^ PA14 cells, in 100 µl of saline, were injected as and described above. Seven days post-BI, CFU counts were assessed in 5–6 mice per group from muscle samples obtained from underneath the burn. Samples were homogenized in 1 ml of 1× PBS. The samples were diluted and plated on LB-agar plates containing rifampicin (50 mg/L).

### Open wound model and CFU assays

The open wound mouse model was used as described in detail elsewhere [54] to examine the long-term bacterial presence at the chronic wound site. Briefly, 6-wk-old CD1 male mice (Charles River; Boston, MA) were anesthetized by intraperitoneal injection and their backs were shaved. Under aseptic conditions, a patch of 1.5×1.5 cm skin was surgically removed from the shaved back of each mouse, exposing an area of collagen connective tissue above the panniculus carnosus muscle. On the top of the surgical wound, 7×10^3^ bacterial cells were inoculated with 10 µl of 2-AA (0.22 mg/kg mice) or an equal volume of vehicle. The surface of the wound was dressed with a transparent and semipermeable Tegaderm film to provide protection from non-experimental bacterial contamination and to allow for daily inspection of the wound. The mice were monitored for 11 d postinfection and CFU counts were performed in 6 mice per group. Tissue samples from the infected area of each mouse were homogenized in 1 ml of PBS, diluted, and plated on LB-agar plates containing rifampicin (50 mg/L).

### Lung infection model and lung biopsy

We assessed the effects of 2-AA in a neonatal mouse lung infection model [55] relevant to CF. A minimum inoculum of 1.5×10^5^ PA14 cells/animal is 100% lethal in this model. Mice were pretreated with 2-AA 4 d before being infected and then sacrificed 24 h, 48 h, or 72 h postinfection. The specimens were fixed in 10% buffered formalin overnight and then, following a dehydration sequence, embedded in paraffin blocks and sectioned into 6-µm-thick sections. The sections were stained with hematoxylin & eosin (H-E) and evaluated by light microscopy. Lung histopathology was assessed and the cytoarchitecture of the infected animals was compared to that of controls.

### *In vivo* cytokine analysis

Blood was collected 24 h post-BI from 4 mice in each group by cardiac heart puncture. Serum was isolated and assayed by multiplex sandwich enzyme-linked immunosorbent assay (ELISA) (Quansys Biosciences, UT) containing nine antibodies (against IL-1α, IL-1β, IL-2, IL-4, IL-6, IL-10, IL-12p70, TNF-α, and IFN-γ). The plate was imaged by cooled CCD camera and integrated density values of the standard spots were used to generate standard curves for the assayed proteins, and density values of the unknown samples were calculated. This service was provided by Quansys Biosciences, USA.

### Cell culture

A Raw 264.7 murine macrophage cell line (American type culture collection, Bethesda, MD) was maintained in Iscove's modified Dulbecco's medium (IMDM, Gibco) supplemented with 10% fetal bovine serum (Gibco) containing penicillin/streptomycin and gentamycin (Gibco) in the presence of 5% CO_2_ at 37°C. The cells were seeded in T-75 tissue culture flasks (Falcon, USA) and used between passages 2 and 3.

### Preparation of stably transfected cells with luciferase reporter plasmid

RAW 264.7 cells (5×10^5^ cells) were seeded in 60-mm dishes; 24 h later, a mixture of Lipofectamine LTX and Plus reagent (Invitrogen, Grand Island, NY) and 2.5 µg of pGL4.-NF-kB luciferase plasmid (a gift from Dr. Xavier's Lab, MGH, Boston) were added and then incubated for 6 h in serum-free medium. The cells were then cultured in medium supplemented with serum for an additional 72 h prior to being subjected to further analysis. Medium containing 250 µg/ml hygromycin B (Roche Applied Science, Basel, Switzerland) was used to select stable transfectants.

### 2-AA cell treatment

Stable RAW 264.7 cells carrying the NF-κB luciferase plasmid were plated at a density of 10^5^/ml in 24-well plates and grown overnight at 37°C in 5% CO_2_. Cells in the treatment groups were pretreated with 0.8 mM 2-AA for 48 h, and then 2-AA treated or non-treated cells were washed with PBS and kept in fresh medium. At various times, as indicated, the cells were stimulated with 0.2 mM, 0.4 mM, or 2 mM 2-AA. Similarly, cells were pretreated with 4-AA (0.8 mM), LPS (100 ng/ml, Sigma-Aldrich), or PGN (100 ng/ml, Sigma-Aldrich) for 48 h. After 48 h, the non-treated or 4-AA/LPS/PGN pretreated cells were stimulated with 4-AA (0.2 mM, 0.4 mM, or 2 mM), LPS (1 ng/ml), or PGN (10 ng/ml), respectively.

### Luciferase assay

After treatment, the stable Raw 264.7 cells were washed with PBS. The cells were then lysed in the luciferase cell culture lysis buffer provided with the Luciferase Assay Kit (Promega, Madison, WI). After a brief vortexing, whole cell lysates were centrifuged at 4°C for 2 min at 12,000 rpm. Supernatant was collected and 20–30 µl of the collected supernatant was added to 60–80 µl of luciferase substrate. Luminescence was measured in a Tecan F200 automated plate reader (Infinite F200, Tecan Group Ltd, Männedorf, Switzerland) and expressed in relative light units (RLU); each luciferase assay substrate reading was taken alone and then with lysate. The value obtained for the luciferase assay substrate without lysate was subtracted from the respective RLU value for each lysate with luciferase assay reagent. The total protein concentration in each lysate was determined with a Bradford assay kit (Thermo Scientific, USA) and subsequently used to normalize the luciferase activity data. Each assay was done in triplicate within each trial and each trial was repeated three times.

### Western blot analysis

Cellular extracts were prepared in RIPA buffer (Cell Signaling Technology, Boston, MA). Twenty micrograms of total protein were added to Lamemli buffer, boiled for 5 min, resolved by SDS-12% polyacrylamide gel electrophoresis (PAGE) in Tris/glycine/SDS buffer (25 mM Tris, 250 mM glycine, 0.1% SDS), and blotted onto PVDF membranes (Bio-Rad, Hercules, CA). The membranes were blocked for 2 h in TBS-T (20 mM Tris-HCL, 150 mM NaCl, 0.1% Tween20) containing 5% non-fat milk. The membranes were then washed three times in TBS-T and probed overnight with rabbit antibodies specific for phospho-NF-κB p65, phospho-extracellular regulated kinase (ERK)1/2, phospho-p38, phospho-c-JUN N-terminal kinase (JNK)1/2 (Cell Signaling Technology), NF-κB p65, Iκ-Bα, IκB-β, or phospho-c-EBPβ (Santa Cruz Biotechnology, Inc., Santa Cruz, CA) at a dilution of 1∶1,000, and mouse anti-β-actin (Santa Cruz Biotechnology, Inc) at a dilution of 1∶2,000. Following three washes in TBS-T, the membranes were incubated with secondary horse-radish peroxidase (HRP)-conjugated goat anti-rabbit IgG (Santa Cruz Biotechnology, Inc) or goat anti-mouse IgG secondary antibodies (Promega, Madison, WI), respectively, and then washed five times in TBS-T. The bands were detected using SuperSignal West Pico Chemiluminescent Substrate (Thermo Scientific, Rockford, IL), according to the manufacturer's instructions.

### IP

For protein association assays, 100-µg aliquots of cytosolic extracts were subjected to IP in TNT buffer [20 mM Tris-HCl, pH 7.5, 200 mM NaCl, Triton ×100, 0.1 M phosphatase inhibitor cocktails 1 and 2 (ingredients from Sigma-Aldrich)]. IP was conducted at 4°C for 3 h with 2 µg of anti-c/EBPβ or 2 µg of p65 (Santa Cruz Biotechnology) and 50 µl of Pierce protein A/G agarose beads (Thermo Scientific). After washing three times with PBS, the precipitated proteins were analyzed by PAGE and western blotting.

### Measurement of TNF-α, IFN-γ, and TGF-β by ELISA

TNF-α, IFN-γ, and TGF-β protein levels in culture supernatants were measured by ELISA using the Quantikine mouse TNF-α and TGF-β kits (R & D Systems, Minneapolis, MN) and an OptEIA mouse IFN-γ kit (BD Biosciences Pharmingen, San Diego, CA) according to the manufacturers' instructions. Briefly, the culture supernatants were added to anti-human TNF-α monoclonal antibody-coated ELISA plates and incubated for 2 h at room temperature. Following four washes, the detector molecules (HRP-conjugated streptavidin and biotinylated anti-human TNF-α) were added to plates and incubated for 2 h at room temperature. The assay was developed with tetramethyl benzidine (TMB) substrate reagent. Following a 20-min incubation at room temperature, the absorbance at 450 nm was determined using a Sunrise plate reader (Tecan Group Ltd, Männedorf, Switzerland). For the TGF-β and IFN-γ assays, we followed the same procedure as described above.

### NF-κB p65/p50 binding assay

Nuclear extracts were obtained from cells at various time points using a Nuclear Extract kit (Active Motif, Carlsbad, CA). Briefly, the cells were scraped in the presence of phosphatase inhibitors into a hypotonic buffer and allowed to swell on ice, before being treated with the non-ionic detergent nonidet-P40 and centrifugation (4°C, 12000 rpm, 15 min). The pellet was resuspended in nuclear lysis buffer, gently rocked for 30 min at 4°C, and centrifuged same as above. The Bradford protein assay was performed on the resultant supernatants (nuclear extracts).

p65 and p50 nuclear binding assays were performed using a NF-κB p65/p50 TransAM transcription factor assay kit (Active Motif, Carlsbad, CA) according to the manufacturer's protocol. Briefly, the wells of a 96-well plate were pretreated with the NF-κB consensus sequence oligonucleotides, and 40 µl of binding buffer was added to the wells; 2-µg nuclear extract aliquots were brought to a mass of 10 µg with lysis buffer and then added to the wells. A 1-µg aliquot of the provided Jurkat nuclear extract was used as a positive control. Following 1 h of incubation, the wells were washed three times with TBS-Tween. Primary antibody was diluted 1∶1500, added to the wells, and incubated for 1 h. Following three washes, secondary HRP-conjugated anti-rabbit antibody was diluted 1∶1000 and added to the wells, incubated for 1 h. After four washes, the developing solution provided in the kit was added. The development was allowed to proceed for 5 min before the reaction was stopped with addition of the kit's stop solution. The absorbance was read on a spectrophotometer at 450 nm with a reference wavelength of 655 nm in a Sunrise plate reader (Tecan Group Ltd, Männedorf, Switzerland)

### MTT assay for cell cytotoxicity

The cytotoxicity of cells treated with 2-AA, 3-AA or 4-AA was measured by MTT assay. MTT (3-[4, 5-dimethyl-2-thiazolyl]-2, 5-diphenyl-2*H*-tetrazolium bromide; Sigma-Aldrich) stock solution (5 mg/ml PBS) was further diluted 1∶5 in PBS. Two hundred microliters of this working solution was added to the cells in a 96-well culture plate for 2 h at 37°C under 5% CO_2_. The dissolved MTT was allowed to convert to insoluble purple formazan via mitochondrial activity in the cells during the 2-h incubation. The supernatant was then removed and the cells were lysed for 10 min with 95% isopropanol-5% formic acid. Absorbance of converted dye was measured at 555 nm, with 690 nm as the reference wavelength using a Sunrise plate reader (Tecan Group Ltd, Männedorf, Switzerland).

### Pharmacological inhibitors for signaling inhibition assay

To investigate the dependence of c/EBPβ activation on ERK1/2, 2-AA pretreated cells were incubated PD98059 (Sigma-Aldrich) for 60 min and stimulated with 2-AA for different time periods. For the cell signaling inhibition assay, cells were treated with NF-κB inhibitors caffeic acid phenethyl ester (CAPE) (1.5 µM, Sigma-Aldrich) or MG-132 (1 µM, Sigma-Aldrich) along with 2-AA. Cells were treated actinomycin D (1 µM, Sigma-Aldrich) during the 2-AA pre-treatment period.

### Statistics

Wherever applicable, at least three independent experiments were performed, and the data were analyzed using the Student's t test or a one-way analysis of variance (ANOVA). The animal data were analyzed using the Kaplan-Meier survivability test. Bacterial CFU counts were analyzed using the Kruskal-Wallis non-parametric test. P values≤0.05 were considered statistically significant.

## Supporting Information

Figure S1 **2-AA activates NF-κB pathways and pro-inflammatory cytokines in mouse macrophages.** (A) Mouse macrophages were incubated with 0.2 mM, 0.4 mM, or 2 mM 2-AA for the indicated time periods, and NF-κB activation was monitored by luciferase assays. The results are expressed as fold change compared to control cells. Mean values calculated from three replicate experiments are depicted with SD error bars. Macrophages were stimulated with 0.2-mM or 0.4-mM 2-AA at the indicated time points. (B) IFN-γ and (C) TNF-α secretion was measured in cell supernatants by ELISA. Mean values calculated from three replicate experiments are depicted with SD error bars. *p<0.05 vs. naïve (Student's t test).
(TIF)

Figure S2 **Structures of 2-AA, the 2-AA metabolite 3OH-2-AA, and the 2-AA analogs 4-AA, 2NA, and MA.**
(TIF)

Figure S3 **Effects of 2-AA on viability of mouse macrophages.** MTT assay measuring cell viability in mouse macrophage cells after treatment with 0.2 mM, 0.4 mM or 0.8 mM 2-AA for different time points, as indicated in the figure. SDs (vertical bars) were calculated from three replicate experiments.
(TIF)

Figure S4 **2-AA pretreated macrophages are broadly hyporesponsive to pathogen associated molecules.** Macrophages were pretreated with 2-AA (0.8 mM), LPS (100 ng/ml), or PGN (100 ng/ml) for 48 h and then stimulated with LPS (1 ng/ml) or PGN (10 ng/ml) for 2 h. Activation of NF-κB (expressed as fold change over background) upon stimulation with LPS or PGN is shown. Mean values calculated from three replicate experiments are depicted with SD error bars.
(TIF)

Figure S5 **2-AA modulates NF-κBp65, p38, JNK, and ERK phosphorylation in 2-AA pretreated mouse macrophages.** Cells were pretreated with 2-AA (2-AA Pre) or medium only (No Pre) for 48 h and subsequently stimulated with 2 mM 2-AA for the indicated time periods. Western blotting of cellular extracts with phospho-specific antibodies was used to reveal the effects of 2-AA pretreatment on phosphorylation of (A) NF-κB p65, (B) p38, (C) JNK1/2, and (D) ERK1/2 following 2-AA (2 mM) stimulation. Loading was normalized relative to mouse β-actin.
(TIF)

Figure S6 **4-AA pretreatment does not alter activation of AP-1 in macrophages upon 4-AA stimulation.** A TransAM AP-1 transcription factor assay after a 48 h pretreatment with 4-AA (0.8 mM) followed by stimulation with 4-AA, showing binding of c-Fos (A) and c-Jun (B) to the AP-1 promoter. Mean values calculated from three replicate experiments are depicted with SD error bars (p<0.05, Student's t test).
(TIF)
