# Supplementary figures and images for: The Quorum Sensing Volatile Molecule 2-Amino Acetophenon Modulates Host Immune Responses in a Manner that Promotes Life with Unwanted Guests
Source: PLoS Pathog. 2012 Nov 15;8(11):e1003024. doi: 10.1371/journal.ppat.1003024 (PMC3499575; doi:10.1371/journal.ppat.1003024)

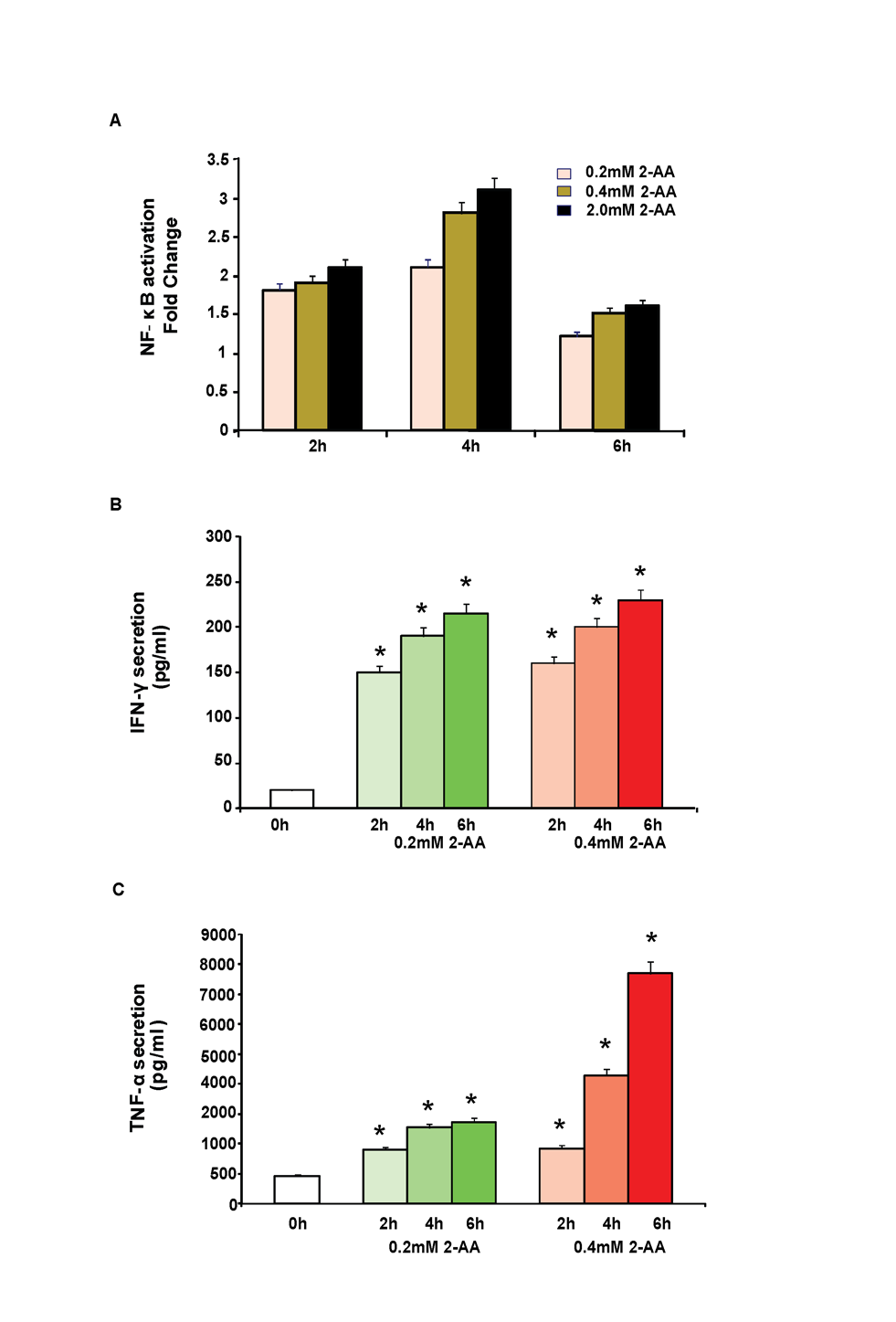

Supplement: Figure S1 — 2-AA activates NF-κB pathways and pro-inflammatory cytokines in mouse macrophages. (A) Mouse macrophages were incubated with 0.2 mM, 0.4 mM, or 2 mM 2-AA for the indicated time periods, and NF-κB activation was monitored by luciferase assays. The results are expressed as fold change compared to control cells. Mean values calculated from three replicate experiments are depicted with SD error bars. Macrophages were stimulated with 0.2-mM or 0.4-mM 2-AA at the indicated time points. (B) IFN-γ and (C) TNF-α secretion was measured in cell supernatants by ELISA. Mean values calculated from three replicate experiments are depicted with SD error bars. *p<0.05 vs. naïve (Student's t test). (TIF) [file ppat.1003024.s001.tif]

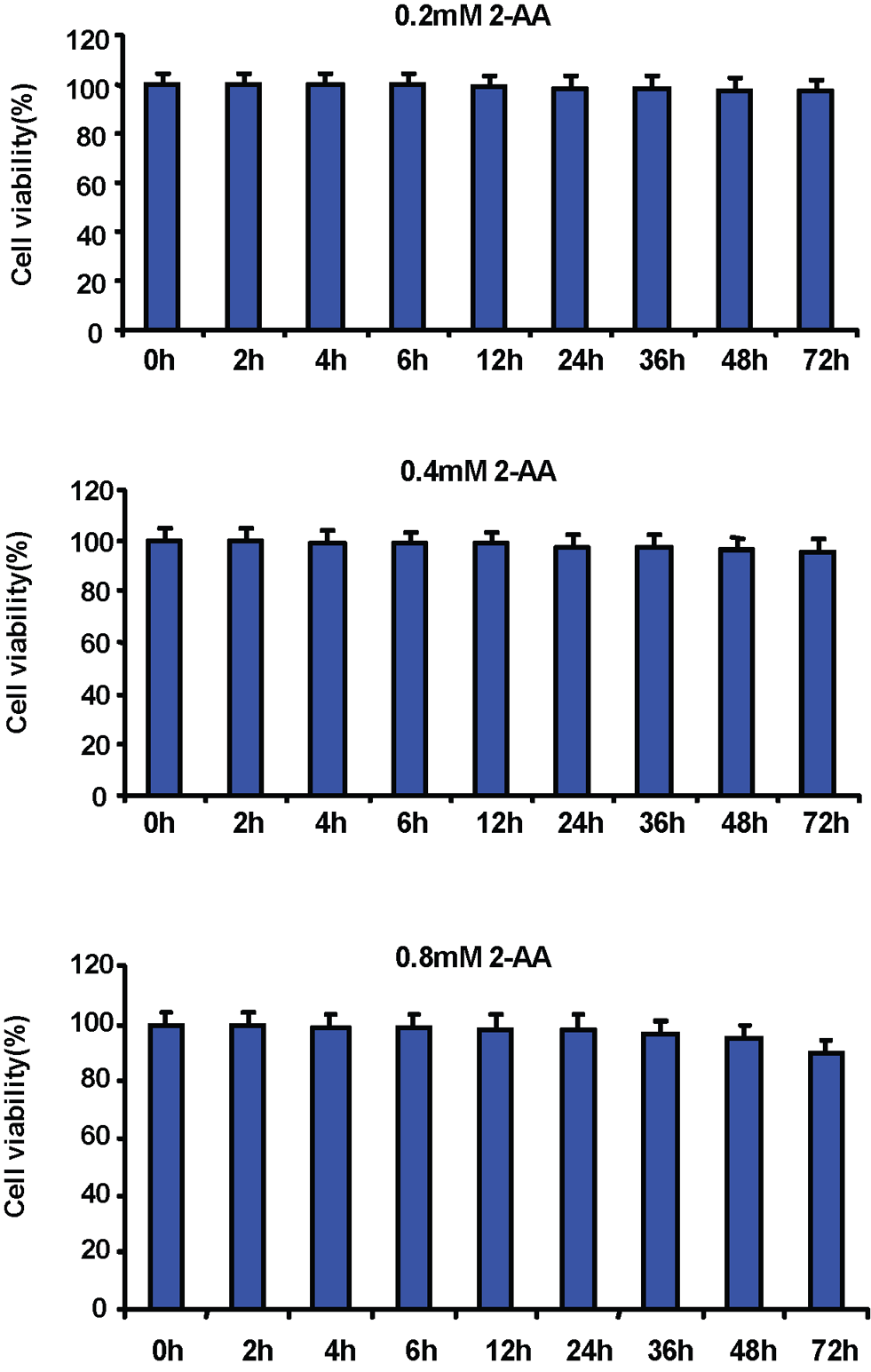

Supplement: Figure S3 — Effects of 2-AA on viability of mouse macrophages. MTT assay measuring cell viability in mouse macrophage cells after treatment with 0.2 mM, 0.4 mM or 0.8 mM 2-AA for different time points, as indicated in the figure. SDs (vertical bars) were calculated from three replicate experiments. (TIF) [file ppat.1003024.s003.tif]

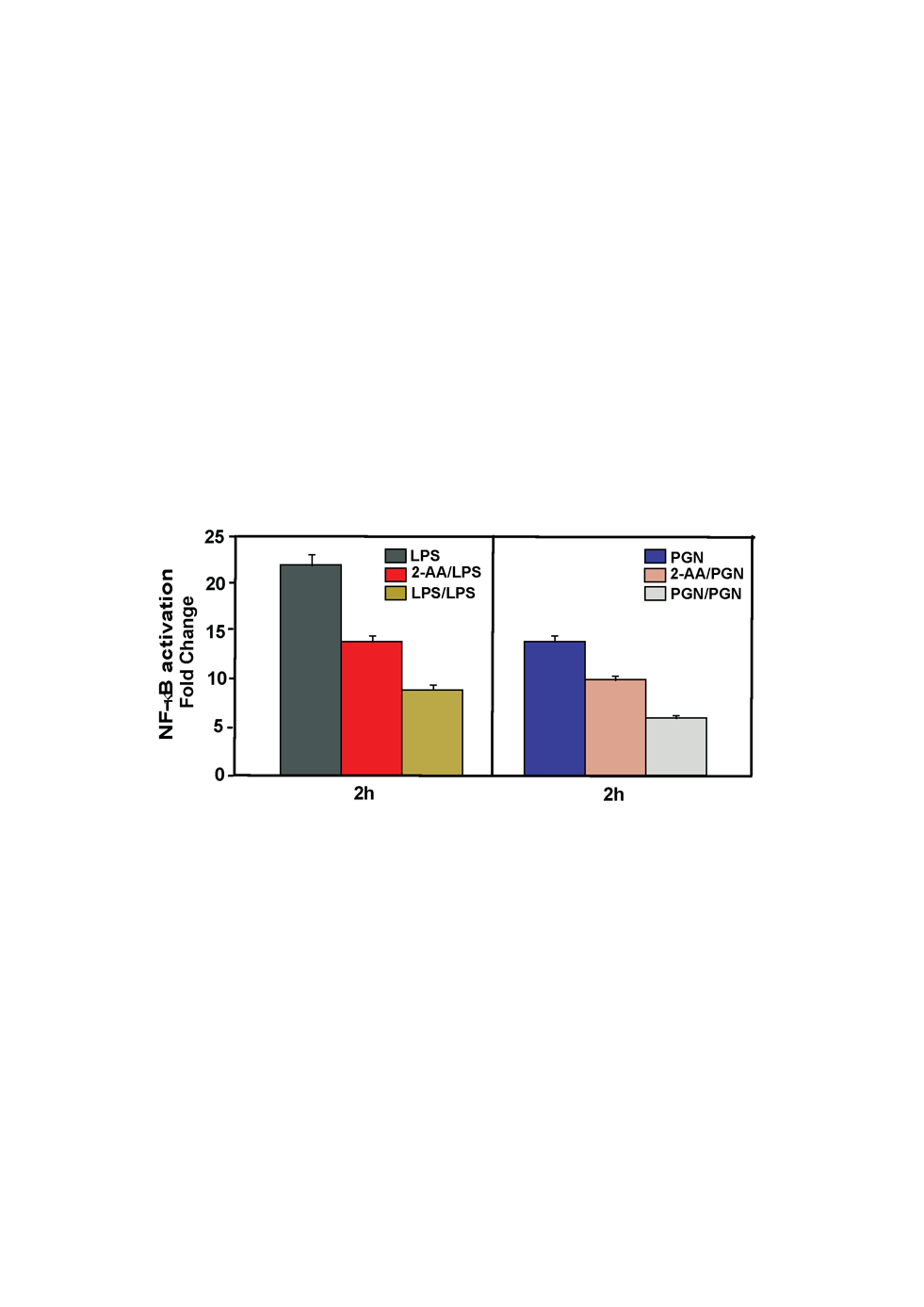

Supplement: Figure S4 — 2-AA pretreated macrophages are broadly hyporesponsive to pathogen associated molecules. Macrophages were pretreated with 2-AA (0.8 mM), LPS (100 ng/ml), or PGN (100 ng/ml) for 48 h and then stimulated with LPS (1 ng/ml) or PGN (10 ng/ml) for 2 h. Activation of NF-κB (expressed as fold change over background) upon stimulation with LPS or PGN is shown. Mean values calculated from three replicate experiments are depicted with SD error bars. (TIF) [file ppat.1003024.s004.tif]

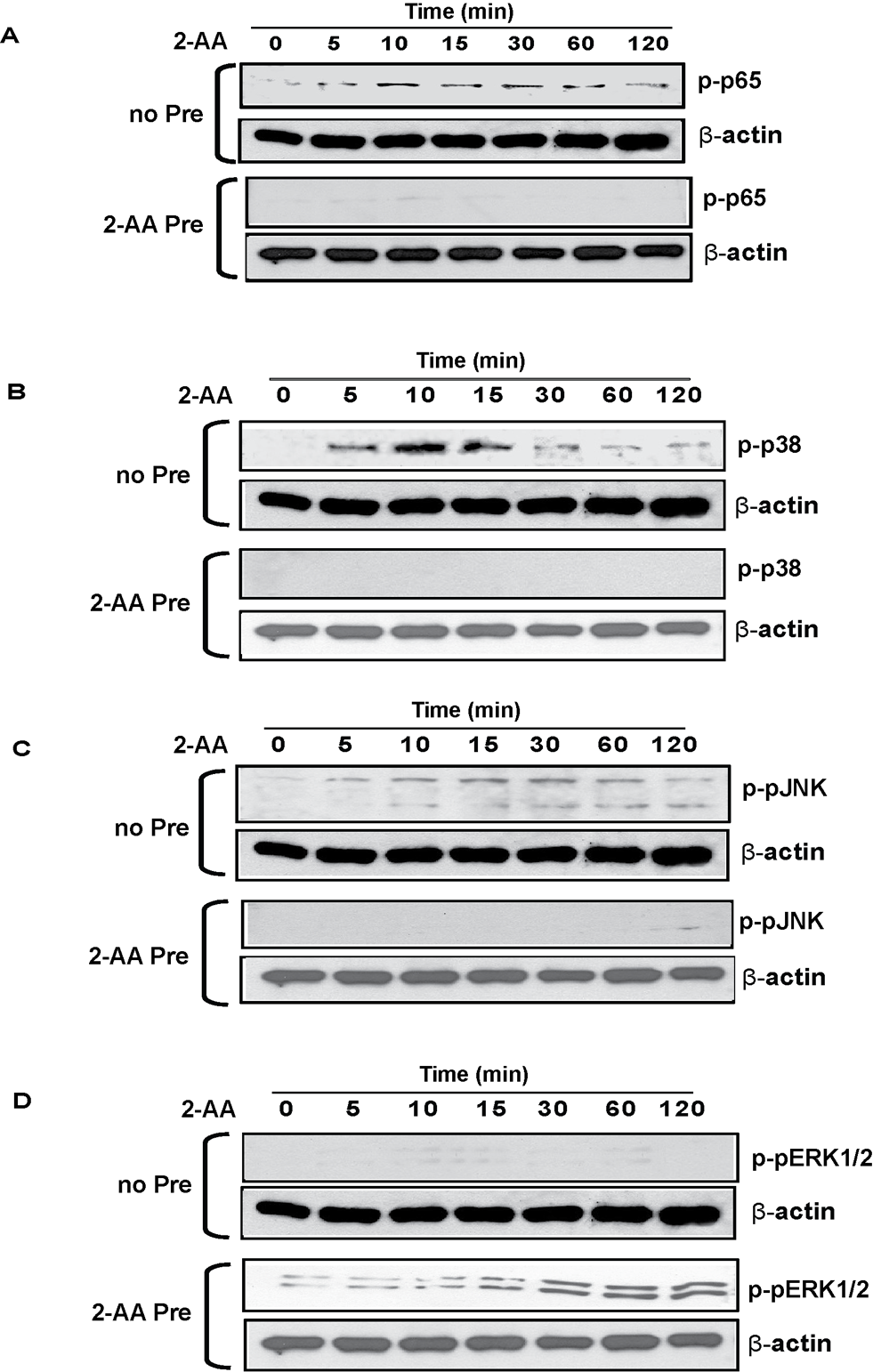

Supplement: Figure S5 — 2-AA modulates NF-κBp65, p38, JNK, and ERK phosphorylation in 2-AA pretreated mouse macrophages. Cells were pretreated with 2-AA (2-AA Pre) or medium only (No Pre) for 48 h and subsequently stimulated with 2 mM 2-AA for the indicated time periods. Western blotting of cellular extracts with phospho-specific antibodies was used to reveal the effects of 2-AA pretreatment on phosphorylation of (A) NF-κB p65, (B) p38, (C) JNK1/2, and (D) ERK1/2 following 2-AA (2 mM) stimulation. Loading was normalized relative to mouse β-actin. (TIF) [file ppat.1003024.s005.tif]

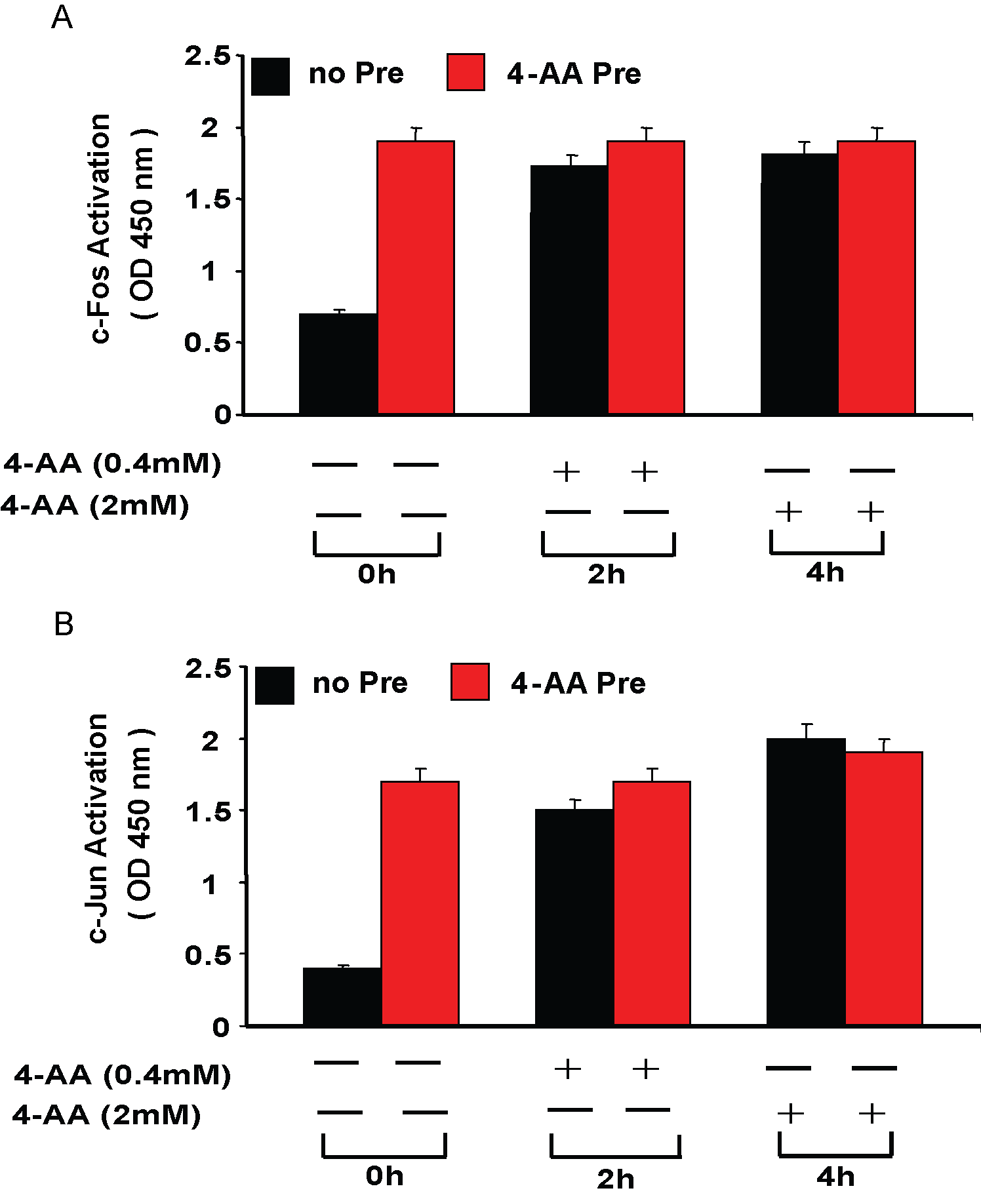

Supplement: Figure S6 — 4-AA pretreatment does not alter activation of AP-1 in macrophages upon 4-AA stimulation. A TransAM AP-1 transcription factor assay after a 48 h pretreatment with 4-AA (0.8 mM) followed by stimulation with 4-AA, showing binding of c-Fos (A) and c-Jun (B) to the AP-1 promoter. Mean values calculated from three replicate experiments are depicted with SD error bars (p<0.05, Student's t test). (TIF) [file ppat.1003024.s006.tif]
